# Explosive utilization efficiency enhancement: An application of machine learning for powder factor prediction using critical rock characteristics

**DOI:** 10.1016/j.heliyon.2024.e33099

**Published:** 2024-06-19

**Authors:** Blessing Olamide Taiwo, Angesom Gebretsadik, Hawraa H. Abbas, Mohammad Khishe, Yewuhalashet Fissha, Esma Kahraman, Ahsan Rabbani, Adams Abiodun Akinlabi

**Affiliations:** a Department of Mining Engineering, Federal University of Technology, Akure, Nigeria; b Department of Resources Engineering, Graduate School of Engineering, Division of Sustainable Resources Engineering, Hokkaido University, Sapporo, 001-0015, Japan; c Department of Mining Engineering, Aksum University, 7080, Aksum, Tigray, Ethiopia; d College of Information Technology Engineering, Al-Zahraa University for Women, 56001, Karbala, Iraq; e Department of Electrical and Electronics Engineering, University of Kerbala, Karbala, 56001, Iraq; f Department of Electrical Engineering, Imam Khomeini Naval Science University of Nowshahr, Nowshahr, Iran; g Innovation Center for Artificial Intelligence Applications, Yuan Ze University, Taiwan; h Applied Science Research Center, Applied Science Private University, 11937, Amman, Jordan; i Department of Geosciences, Geotechnology and Materials Engineering for Resources, Graduate School of International Resource Sciences, Akita University, Japan; j Department of Mining Engineering, Cukurova University, Adana, 01250, Turkey; k Department of Civil Engineering, Sai Nath University, Ranchi, India

**Keywords:** Explosive, Mining, Powder factor, Machine learning, Small scale mine

## Abstract

Maximizing the use of explosives is crucial for optimizing blasting operations, significantly influencing productivity and cost-effectiveness in mining activities. This work explores the incorporation of machine learning methods to predict powder factor, a crucial measure for assessing the effectiveness of explosive deployment, using important rock characteristics. The goal is to enhance the accuracy of powder factor prediction by employing machine learning methods, namely decision tree models and artificial neural networks. The analysis finds key rock factors that have a substantial impact on the powder factor, hence enabling more accurate planning and execution of blasting operations. The analysis uses data from 180 blast rounds carried out at a dolomite mine in south-south Nigeria. It incorporates measures such as root mean square error (RSME), mean absolute error (MAE), R-squared (R^2^), and variance accounted for (VAF) to determine the best models for predicting powder factor. The results indicate that the decision tree model (MD4) outperforms alternative approaches, such as artificial neural networks and Gaussian Process Regression (GPR). In addition, the research presents an efficient artificial neural network equation (MD2) for estimating the values of optimum powder factor, demonstrating outstanding blasting fragmentation. In conclusion, this research provides significant information for improving the accuracy of powder factor prediction, which is especially advantageous for small-scale blasting operations.

## 1 Introduction

In mining, construction, and demolition, blasting operations use explosives to break up rock formations and clear ground. Blasting is crucial in the mining industry as it allows access to rich minerals or resources by opening up rock formations [1]. Similar to mining, construction uses blasting to remove large amounts of rock or terrain to create space for buildings [2]. One cannot overstate the importance of blasting operations in mining and construction. Blasting enables the effective exploitation of resources by splitting massive rock formations into smaller, more manageable rock pieces that are readily transportable and processed. Additionally, blasting activities are necessary to make apertures and alter the topography to meet construction requirements, including digging tunnels for infrastructure projects or laying building foundations. Furthermore, blasting is a critical component of mining and construction activities' increased productivity and decreased safety concerns [3,4]. Well-designed blasting patterns can reduce safety risks and increase production rates. Blasting works generate seismic effects that need to be evaluated to assess their impact on structures and the geological environment [5]. To minimize any negative effects on the environment, such as air pollution and ground vibrations, and to protect surrounding structures and personnel while optimizing fragmentation, blasting operations must be managed appropriately. A consistent technique of computation that takes into consideration the unique characteristics of powder factor, explosive utilization, and rock properties is required to increase the efficacy of blasting [6]. The idea of the powder factor, which refers to the amount of explosives needed to fracture a specific volume of rock, is essential for optimizing the usage of explosives in blasting operations [7]. The primary purpose of blasting is to reduce rock size and facilitate transportation from the mine to the processing facility. Different powder factors can affect blast fragmentation and ultimately impact production output, safety, and profitability [8]. Mohamed [9] has observed the influence of rock mass characteristics, blasting material, and blasting geometry. Geological variables, rock qualities, and blast design parameters. Rock type and structure affect how explosives interact with rock mass, affecting fragmentation. Rock's response to explosive energy depends on density, hardness, and brittleness, requiring specialized blasting methods. Blast design characteristics, including hole diameter, spacing, and stemming, help distribute explosive energy for optimal fragmentation. These parameters determine the powder factor needed for blasting results, emphasizing the need for thorough planning and execution.

Small-scale mine blasting operations provide several difficulties, particularly in guaranteeing safety, effectiveness, and environmental accountability [10]. Previous literature has identified many primary challenges, such as effectively regulating blast vibrations to avoid structural damage, limiting flyrock to safeguard neighboring infrastructure, and controlling airblasts to mitigate potential injury to personnel. Furthermore, it is critical to carefully select powder elements that are consistent with the rock's specific properties. The limited access to resources for small-scale mine operators may impede their capacity to obtain equipment and training, which significantly increases the likelihood of worsening safety concerns. In order to mitigate these issues, it is crucial to implement thorough risk assessments, hire competent workers, and utilize advanced blasting procedures. However, it has been observed that Africa and many small-scale miners worldwide do not prioritize these measures. Choosing the optimal powder factor for small-scale mine blasting operations poses difficulties because it necessitates striking a balance between efficiency and safety. Opting for a powder factor that is too low can result in inadequate fragmentation, which can lead to expensive secondary blasting and higher operational costs [11]. On the other hand, choosing a high powder factor might create safety hazards, such as an abundance of flyrock or ground vibration, particularly in the limited areas commonly found in small-scale mines. Furthermore, the presence of different rock characteristics and geotechnical conditions adds complexity to the process of selecting the appropriate powder factor. In order to achieve the ideal equilibrium, it is necessary to engage in careful and detailed planning, conduct precise evaluations of geological conditions, and continuously monitor operations to ensure both efficiency and safety. A multitude of factors, such as geological properties, rock characteristics, and blast design parameters, intimately influence the prediction of powder factors in small-scale mine blasting operations. Geological factors, such as the type and structure of the rock, determine how explosives interact with the rock mass, which in turn affects the consequences of fragmentation. Density, hardness, and brittleness of a rock determine its response to explosive energy. This requires the use of customized blasting techniques. The blast design characteristics, including hole diameter, spacing, and stemming, are crucial in efficiently spreading explosive energy to achieve the best possible fragmentation. The powder factor required to accomplish desired blasting results is influenced by these parameters collectively, emphasizing the significance of a thorough understanding and meticulous consideration in the design and execution of blasting operations.

Empirical theories and formulas are utilized to forecast the powder factor in rock blasting. Taiwo et al. [12] developed an artificial neural network (ANN) model to enhance the efficiency of explosive utilization in small-scale quarries, resulting in a notable level of prediction precision. Thangavel and Samui utilized an ANN model to predict the average particle size resulting from blast fragmentation, considering multiple input variables [13]. Jiang [14] introduced a model that utilizes the Gaussian process (GP) and the differential evolution algorithm (DE) to forecast blast-induced ground vibration (BIGV). The model incorporates the powder factor as an input variable. Sanchidrián developed a fragmentation-energy fan model to examine the relationship between fragmentation and powder factor, achieving high levels of prediction accuracy [15]. These studies demonstrate the use of empirical models and formulas to forecast powder factors, as well as their impact on blast fragmentation and ground vibration. Several drawbacks constrain the conventional methods for predicting powder factor in rock blasting, including their reliance on oversimplified assumptions, limited accuracy in heterogeneous geological environments, and inability to account for changing site conditions [7,16]. These approaches often overlook the influence of mixed geological formations on the powder factor [17] and may not adequately evaluate the risk of blasting-induced rock damage [18]. In order to overcome these constraints, it is necessary to employ a more extensive and precise approach that incorporates various characteristics of the rock mass, the materials used for blasting, and the geometry of the blasting process. Additionally, this approach should take into account the specific energy and particle size as defined by comminution theory. Calculating the optimal powder factor for a blasting scenario involves dealing with multiple intricacies and uncertainties. The geological heterogeneity, encompassing diverse rock types and structures, has a profound impact on the interaction between explosives and the rock mass. The fragmentation outcomes are significantly influenced by rock qualities, such as density and hardness, while the optimization of blast design parameters, such as hole diameter and spacing, is essential. The specific characteristics and limitations of the site, along with the uncertainty in predictive models based on historical data, further complicate the procedure. Furthermore, the ever-changing characteristics of blasting activities, the need for safety precautions, and the need for environmental protection necessitate a careful balance between effectiveness and potential hazards reduction. In order to tackle these issues, it is necessary to have a comprehensive grasp of blasting concepts, utilize advanced modeling techniques, and possess practical expertise in order to achieve the best possible blasting results. The use of machine learning approaches, such as ANN, can help optimize explosive use in small-scale quarries [15]. The fragmentation-energy fan model, represented by the Swebrec function, can analyze the dependence of fragmentation on the powder factor and predict fragment sizes with high accuracy [19,20]. Other factors, such as the delay between holes and geotechnical properties of the rock mass, can also influence the powder factor and blasting. Rock mass characterization, using tools like the Schmidt hammer, can aid in determining the optimum powder factor for bench blasting in different rock types [21,22]. The main aims of this research are two-fold: firstly, to improve the accuracy of predicting powder factors in small-scale mine blasting operations by implementing artificial intelligence, and secondly, to optimize the efficiency of using explosives in mining operations. This improvement involves utilizing machine learning methods to create models that can precisely predict powder factors by considering several input parameters, such as geological features, rock qualities, and blast design parameters, unlike available empirical models limited to controllable parameters. The research intends to enhance cost reduction, optimize blasting conditions, and enhance operational efficiency in the mining and construction industries by attaining more accurate estimates of the powder factor.

## 2 Literature review

In their study, Mohamed [9] emphasized the importance of the powder factor in maximizing the efficiency of explosive use during blasting activities. Xu [23] also noted that the definition of specific charge highlights its vital role in assessing the effectiveness of blasting, as it quantifies the quantity of explosive material employed per unit mass of cracked rock. A study has demonstrated that various factors, such as geological variables, rock properties, and blast design parameters, intricately influence the powder factor calculation. Knowledge of the kind and structure of the rock heavily influences an understanding of the performance of explosive energy during blasting. This knowledge plays a crucial role in determining the interaction between explosives and the rock mass, which ultimately affects the subsequent fragmentation. Moreover, Scott's [24] research demonstrated a strong correlation between the response of rock to explosive energy and its density, hardness, and brittleness. Consequently, specialized blasting techniques are necessary. The exact characteristics of blast design, including weight, hole length, spacing, and stemming, play a vital role in efficiently distributing explosive energy to achieve optimal fragmentation. The calculation of the powder factor required for successful blasting outcomes involves a careful assessment of rock characteristics. However, the utilization of artificial intelligence to simulate the rock class and structure and select the most suitable model is crucial in order to prevent the need for extensive planning and execution in blasting operations. We can classify the factors that influence the fragmentation of rocks during blasting as controllable and uncontrollable. In this context, we refer to variables like burden, blast-hole diameter, powder factor, and delay timing as controllable factors. Conversely, we cannot directly control or manipulate uncontrolled factors like hardness factors, joints, and in-situ block size [25]. According to Mulenga [26], the interrelations diagram method showed that controllable causes account for 90 % of the cumulative percentage contribution to rock fragmentation, whereas uncontrollable factors only contribute 10 %. To get the fragmentation you need, you have to make sure that things you can control, like the blast geometry and explosive properties, work with things you can't, like geological conditions and legal limits [27]. Blaster design factors such as burden, spacing, stemming, bench stiffness ratio, and powder factor all have a significant impact on the fragmentation that occurs after a blast [28]. By manipulating these factors, it is possible to achieve the most favorable fragmentation, resulting in mean fragment sizes that can vary between 50 % and 200 % [28,29]. Numerous articles have analyzed the relationship between controllable factors during an explosion and the subsequent rock fragmentation. Taiwo [11] discovered that the charge load ratio, stiffness ratio, maximal instantaneous charge, and specific charge all exert an impact on the particle size distribution of fragmented rocks. Shehu [25] discovered a strong link between blast design parameters and the average size of fragments. Specifically, they found that the stiffness ratio and powder factor had high correlation coefficients. Figueiredo et al. employed the Porta Metrics TM tool to examine fragmentation and determined that it is a dependable instrument for assessing blast design [30]. Li Qingxiang and his colleagues conducted a quantitative analysis of the impact of blasting settings on rock fragmentation. They also developed a control model using a double-hidden-layer BP neural network, as described in the study by Li [31]. These studies emphasize the significance of blast design factors in attaining the intended rock fragmentation results.

Understanding the classification of rocks and their strength properties is crucial for improving the efficiency of explosive utilization in mining and building activities. Rock mass classification systems, such as RMR (Rock Mass Rating), RQD (Rock Quality Designation), and GSI (Geological Strength Index), provide a systematic approach to evaluating various rock mass characteristics. These assessments can help predict how long subterranean excavations can maintain themselves and determine the specific type of support needed [32]. Moreover, it is essential to comprehend the shear strength and compressive strength of rocks in order to assess the stability and safety of mining structures, as well as to optimize the effectiveness of explosives in quarrying and material extraction [33,34]. Understanding the categorization and durability characteristics of rocks allows for the accurate and effective use of explosives in a variety of mining and construction scenarios. Accurate classification of rock formations facilitates the choice of the appropriate explosive and its practical application during blasting operations. It ensures the accurate distribution of explosive material for each unit of rock, leading to a significant decrease in rock size and transportation needs. Artificial intelligence (AI) models, namely ANN and support vector machine (SVM) techniques, have been integrated with optimization algorithms like particle swarm optimization (PSO), genetic algorithm (GA), and grey wolf optimization (GWO) to develop rock mass classification models that are easy for users to navigate. The models have exhibited remarkable prediction precision and a capacity to effectively generalize across many influencing parameters [12,35]. The models have demonstrated remarkable accuracy, precision, and recall in predicting the categorization of rock masses, allowing for more precise powder factor calculation and parameter selection for blasting design [35]. Multiple parameters associated with the energy of rock fracturing affect the propagation of rock waves. The rock's mechanical properties, including its elastic modulus and Poisson's ratio, greatly influence the shape, amplitude, and frequency of the transmitted waves [36]. The existence of cracks and joints in the rock mass influences wave propagation. The distance and direction of the cracks can alter the waveform, rising time, and wave coefficient of the transmitted and reflected stress waves [37]. Furthermore, the characteristics of faults, such as the angle of inclination and the material filling them, might impact the maximum particle velocity and the propagation of explosive shockwaves [38]. Seismic waves can affect the hydraulic characteristics of fractured rock formations, increasing permeability and altering flow rate patterns [39]. Understanding these connections is critical for assessing rock stability, forecasting rock calamities, and enhancing the precision of rock catastrophe prediction using electromagnetic radiation [40]. The bulk density of a rock is a crucial factor in mining operations due to its impact on the effectiveness of explosive utilization and the fragmentation of the rock during blasting. Research has indicated that the powder factor, which refers to the amount of explosive used per unit of fragmented rock, can be maximized by considering the bulk density of the rock. Researchers have observed that different powder variables produce different levels of blast fragmentation and uniformity index [12,16]. By utilizing comminution theory and work index, predictive techniques have been devised to estimate the powder factor and optimize blasting settings [16,41,42]. These methods seek to minimize expenses by eliminating the need for trial-and-error blasting and enhancing the effectiveness of explosive utilization in mining activities.

Powder factor computation, prediction, and optimization are essential considerations in various industries, such as additive manufacturing and mining. Numerical simulations and ANN models predict and evaluate the quality of powder beds and molten tracks in the field of additive manufacturing [43]. The mining industry employs statistical methods and artificial neural networks to determine the powder factor, taking into account geological conditions, rock properties, and design parameters [8,44]. Additionally, comminution theory and work index are utilized to develop predictive methods for powder factor estimation, reducing the need for trial-and-error blasting [16,41]. These approaches aim to optimize the powder factor, which has a direct impact on production costs, downstream operations, and blast fragmentation size distribution. By accurately predicting and optimizing the powder factor, industries can improve efficiency and reduce costs in their operations. Researchers have devised various empirical models and formulas to forecast powder factors in rock blasting. Leu employed artificial neural networks to develop a model for tunnel blasting, where the designation of rock quality was recognized as a crucial element [45]. In his study, Agyei examined different techniques for estimating powder factor, encompassing empirical and comminution theory modeling as well as machine learning methodologies [7]. Moomivand constructed an empirical fragmentation model that integrated rock mass characteristics, blasthole parameters, and powder factors, resulting in a strong correlation with real-world outcomes [46]. Ghasemi conducted a study that specifically examined the estimation of the distance that flyrock travels in surface mines [47]. The study identified the powder factor as a crucial parameter in this prediction. These studies emphasize the significance of the powder factor in rock blasting and the possibility of using empirical models to enhance its prediction. Researchers have conducted various studies to investigate the relationship between powder components and fragmentation. This research has employed diverse techniques, including ANN modeling, sieving, and statistical analysis, to enhance the efficiency of explosive utilization and forecast the extent of fragmentation. The findings indicate that the most favorable powder factor for small-scale quarries falls within the range of 0.7–0.8 kg/m3 [8]. Sanchidrián [15] have determined that the fragmentation-energy fan model, which relies on powder factor and delay, effectively forecasts fragment sizes. Several techniques have been suggested for estimating it, such as employing the Schmidt hammer to assess rock properties [9,21], utilizing the Bond work index for predictive modeling [16], and taking into account rock and design characteristics [48]. The primary objective is to attain the optimal powder factor to produce desired blasting outcomes, including fragmentation, throw, and ground vibration, while lowering the overall expenses associated with mining operations [48]. Conventional methods for estimating powder factors in rock blasting are subject to several constraints. These methods frequently depend on oversimplified assumptions that may not adequately depict the intricate and diverse geological conditions observed in mining activities [8]. In addition, conventional techniques may fail to consider the dynamic variables of a site, such as alterations in rock characteristics or weather conditions, which can have a substantial effect on the effectiveness of a blast [49]. Inadequate precision in conventional methods can result in inefficient blast designs, leading to escalated expenses and associated hazards [12]. In order to address these constraints, scientists have investigated the application of ANN and optimization methods to enhance the precision of powder factor prediction [50]. Modern methodologies allow for the consideration of a wider range of elements and variables, leading to more accurate and reliable forecasts [51]. Machine learning, an integral element of artificial intelligence, is employed to develop algorithms by analyzing data patterns and historical correlations. The applications of this technology encompass several fields, such as bioinformatics, intrusion detection, information retrieval, game playing, marketing, virus detection, and picture deconvolution [52,53]. Notably, the field of bioinformatics has made substantial progress, particularly in the development of advanced machine-learning methods to tackle the complexities and expenses associated with biological analyses. These applications exemplify the capacity of machine learning to examine intricate datasets, discern trends, and generate precise forecasts. Machine learning has transformed the mining and construction sectors by facilitating the examination of intricate information, the discovery of patterns, and precise forecasting. Machine learning has been utilized in the field of construction for tasks such as site supervision, automatic detection, and intelligent maintenance [23,54]. In the field of mining, mineralogical data has been utilized for exploitation purposes, mainly through the utilization of support vector machines and deep learning models, which are the most often applied techniques [55,56]. Zelinska emphasized the technology's capacity to enhance mining operations, control equipment performance, and ensure environmental security [57]. Despite these advancements, there are still obstacles to overcome, including the need for annotated data and the laborious process of building machine learning systems.

Advanced technologies, including machine learning, play a vital role in improving the efficiency of explosive consumption by predicting powder factors. Agyei and Kahriman [7,16] emphasize the significance of precise powder factor estimation in cost reduction and enhancement of blasting conditions. Agyei focuses on the application of machine learning in estimating powder factors, whereas Kahriman presents a methodology based on the Bond work index. Ohdar and Suzuki [58,59] emphasize the capacity of machine learning to forecast process parameters and analyze data, respectively, within the realm of materials research. These studies collectively show how new technology, especially machine learning, can improve the efficiency of explosive consumption by predicting powder factors.

The objective of this study is to utilize machine learning techniques to improve the accuracy of predicting powder factors. This improvement will be achieved by incorporating Critical Rock characteristics (CRC) input data such as bulk density, rebound hardness value, p-wave velocity, rock grade designation, and burden-to-spacing ratio. These data can be utilized as features in machine learning models to predict the powder factor required for blasting operations. This system is unique because it integrates advanced machine-learning approaches to predict powder variables in blasting operations. The initiative seeks to employ computational techniques to overcome the limitations of conventional empirical models and provide more accurate predictions.

Furthermore, the comprehensive integration of various input data, including geological features and blast design parameters, sets this research apart. By employing a holistic methodology, one can attain a more all-encompassing comprehension of the diverse factors that influence the powder factor. Consequently, this enhances the precision and forecasting capability of the models. The use of advanced technologies and the focus on increasing efficiency show a commitment to innovation and addressing contemporary challenges in the mining and construction sector.

## 3 Field study and lab work

**Fig. 1:**
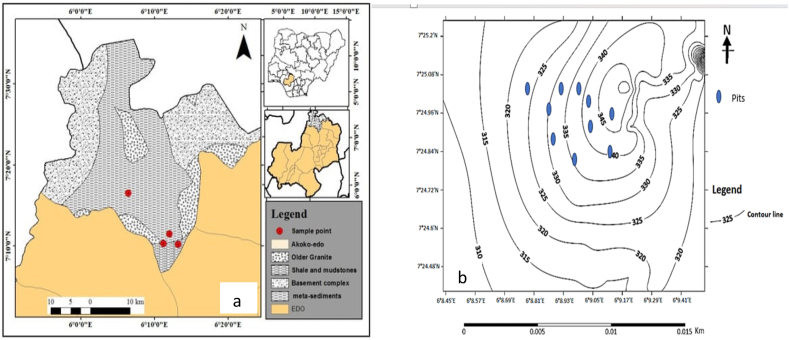
Topographical map of Akoko Edo showing the case study quarry (After [8]).

**Fig. 2:**
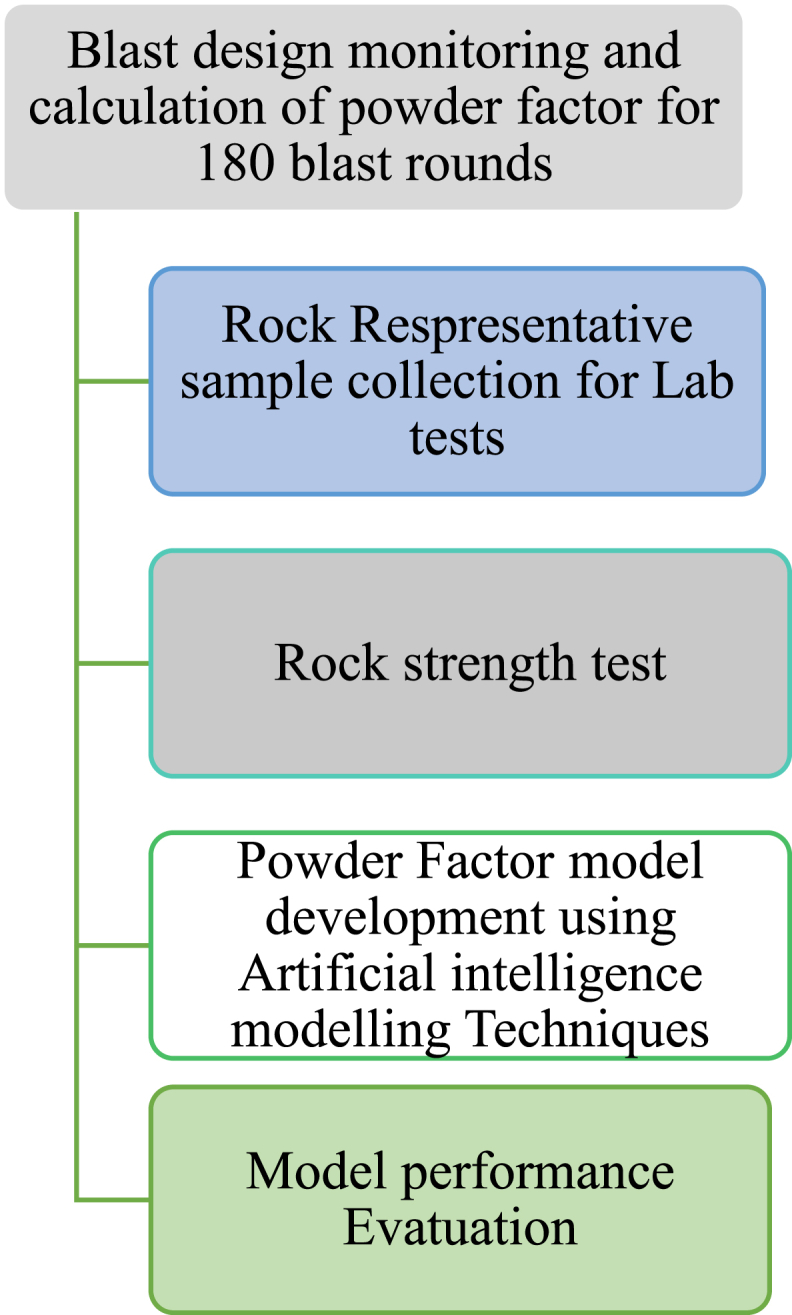
A flow chart of the study objectives.

The case study mine is located in Akoko Edo, Edo State, Nigeria, on a private mineral claim (See Fig. 1a). The area's formation consists of white coarse marble, white dolomite, and clay soil overburden. Fig. 1b displays the Sufa-created topographic map of the mine region. The mine drills blast holes ranging in size from 1.2 to 1.45 m. The mine's rock strength in accordance with the International Society of Rock Mechanics Commission was determined. Each blast round was drilled in a staggered pattern using a 25-mm packaged emulsion gel explosive and a column charge of ammonium nitrate and fuel oil (ANFO). The 1.5-m-tall experimental mine benches were drilled and blasted with a handheld jackhammer of small diametre and 25-mm packed emulsion gel explosive backed by ANFO. The powder factor for 180 blast rounds was computed in the same sequence using the rule of thumb method. For this study, we considered ten pits owned by individual small-scale miners. The strength properties of each 10-pit formation were analyzed using a representative sample from the blast rounds. The International Society of Rock Mechanics Commission's guidelines guided the assessment of the mine's rock strength. Fig. 2 presents the holistic objectives of the study.

### 3.1 Determination of rock strength properties

#### 3.1.1 Determination of uniaxial compression strength

The Uniaxial compressive strength (UCS) test in this study was carried out in accordance with the method suggested by the International Society of Rock Mechanic Commission [60]. The compressive strength of a rock may be regarded as the highest stress that a rock specimen can carry when unidirectional stress is applied, generally in an axial direction, to the ends of a cylindrical specimen [61]. Uniaxial compressive strength test is typically characterized by loading a cylindrical sample with a diameter of approximately 50 mm and a length-to-diameter ratio of 2.5–3.1 axially until the specimen fails. The Uniaxial Compressive Strength was determined using Eq. (1). Basically, the test involves subjecting the rock samples to compression loads until the rock sample fractures after reaching its maximum loading strength.

(1) $$\text{UCS}=\frac{P}{A}$$

where UCS is the uniaxial compressive strength (MPa), P is the applied peak load (kN), and A is the cross-sectional area (m^2^).

#### 3.1.2 Determination of rock bulk density and P-wave velocity

The Rock bulk density in this study was tested in accordance with the International Society of Rock Mechanics Commission. Representative samples of about 300 g were prepared for samples taken from the ten pits. The change in the rock volume during testing was indicated on the measuring cylinder and recorded accordingly. The bulk density was determined using Eq. (2).

(2) $$\text{Bulk}\;\text{density}\;\left(g/{\text{cm}}^{3}\right)=\frac{M}{V}$$

where *M* is the mass of the sample (g), and *V* is the volume displaced (cm^3^).

The standard procedure for measuring Rock P-wave velocity, which involves using a laboratory ultrasonic testing apparatus, was adopted in this study. First, cylindrical rock samples were prepared and saturated to eliminate any air gaps. The ultrasonic transducers were then placed on either end of the sample, and a known frequency signal was transmitted through the rock. The time taken for the P-wave to travel through the sample was recorded. The primary wave velocity was calculated using the formula present in Eq. (3). This procedure adheres to ASTM International standard D2845-17, ensuring accuracy and consistency in rock velocity measurements (ASTM, 2008)

(3) P−wavevelocity=distancetraveled/timetaken.

#### 3.1.3 Determination of rock mass classification

The Rock Mass Rating (RMR) is a geomechanical classification system used to assess the quality of rock masses for engineering purposes. This classification system was developed by Bieniawski [62]; RMR considers six parameters, including uniaxial compressive strength, rock quality designation, spacing of discontinuities, condition of discontinuities, groundwater conditions, and orientation of major joints. Each of these parameters was collected from the study mine and was assigned a weight in accordance with the Bieniawski rating table, and the RMR was calculated by summing these weighted values. The discontinuity properties of the in-situ rock were measured using the window mapping method, and this gives adequate and sufficient information about the investigated area.

### 3.2 Data analysis and visualization

**Fig. 3:**
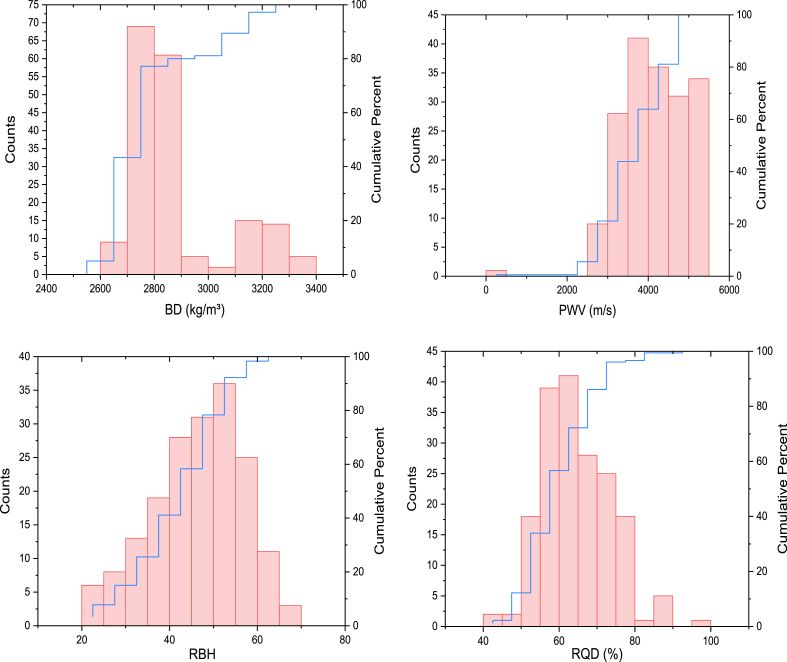

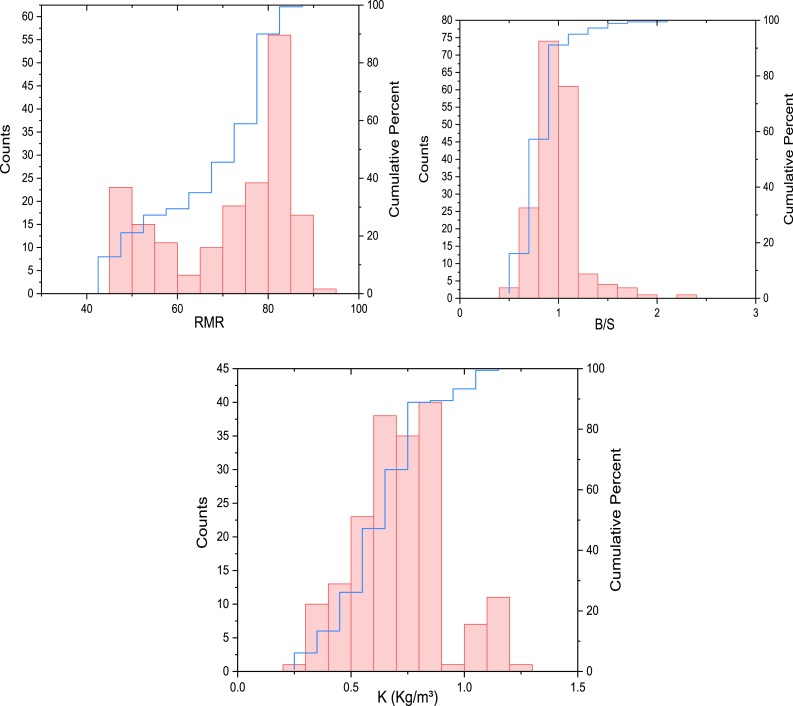
The illustration of the frequency distribution of all variables.

The authors investigated a dataset collected from the case study area to develop accurate predictive models for the powder factor; the dataset consisted of 180 test results from the quarry blasting in Akoko Edo, Nigeria. To understand the data range and distribution, the data analysis was carried out using Origin Pro 2024 software. The study utilized six input parameters, including rock mass rating (RMR), bulk density (BD), rebound hardness value (RBH), P-wave velocity (PWV), rock quality designation (RQD), and burden-to-spacing ratio (S/B) for the prediction of powder factor (K). Fig. 3 provides more clarification on the distribution of each variable based on the frequency plot. The graphical depiction of the data shown in this image was beneficial in detecting any possible anomalies or patterns that might influence the precision of the models.

**Fig. 4:**
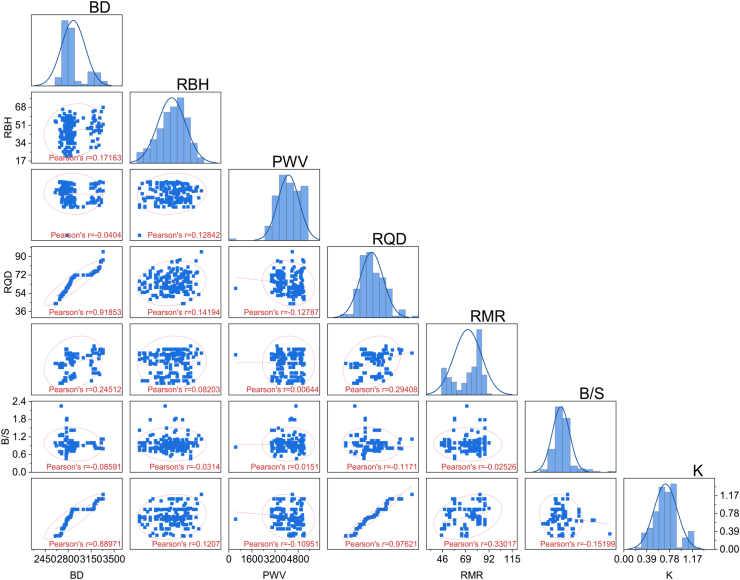
The illustration of a scatter matrix plot of all the variables to elaborate the relationship between the variables.

Fig. 4 demonstrates the relationship between variables in terms of the scatter matrix plot. It illustrates that all variables very strongly correlate with each other. It shows a strong correlation between K and BD with (r = 0.88). The high correlation (r = 0.88) between powder factor and bulk density indicates a robust and reliable link between these variables in blasting operations. Bulk density, which measures the amount of material per unit volume, has a direct impact on how explosives are distributed and how energy spreads within the rock mass during blasting. The result indicated that higher bulk density often suggests a more compact rock, meaning it requires more energy to break apart. This correlation is reflected in a larger powder factor. This correlation emphasizes the significance of comprehending rock density parameters in order to optimize blasting designs and attain the appropriate fragmentation outcomes in mining and quarrying operations.

**Fig. 5:**
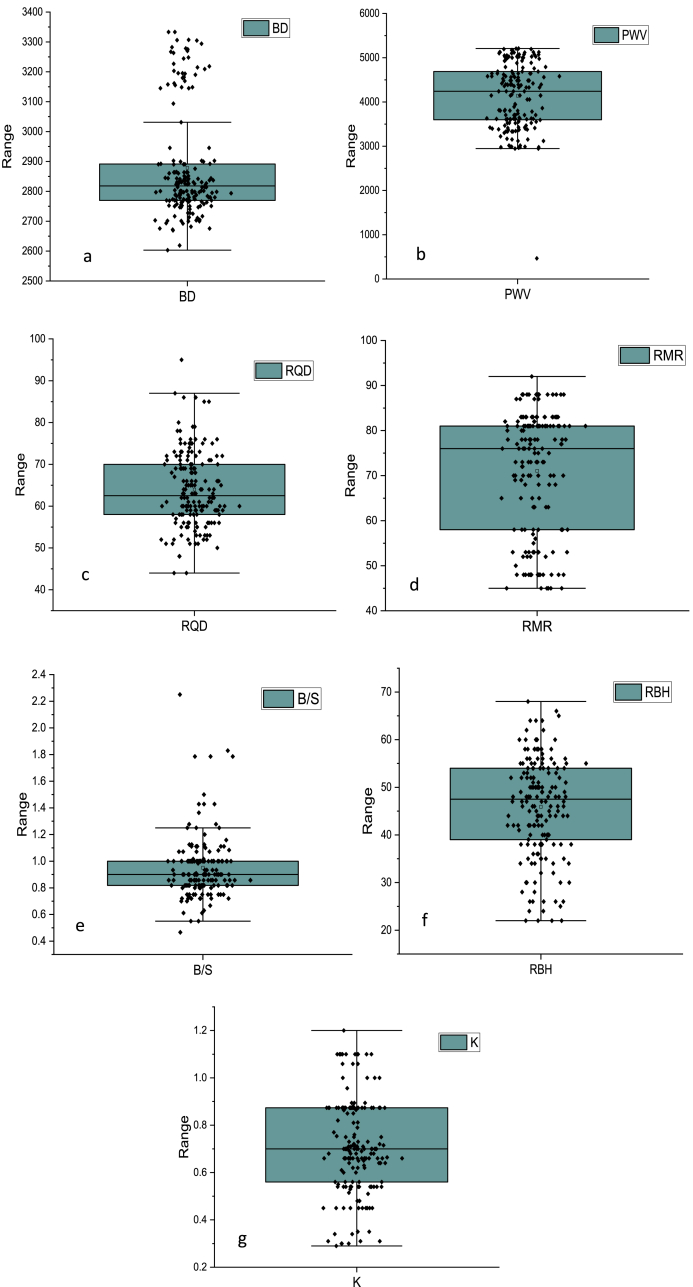
Depiction of violin plots of all variables.

The violin plots of the variables are depicted in Fig. 5a-g. It is helpful in visualizing the distribution of the blasting dataset of this study, especially in terms of quartiles. Quartile _1_ *(*Q_1_) represents the 25th percentile, and Quartile _3_ (Q_3_) represents the 75th percentile. The box-in-a-box plot spans from Q_1_ to Q_3_. For RBH, the data points are highly scattered in Q_1_ and Q_3_, with a range of 23–66. Similarly, the output K data set is highly distributed in a range of 0.3 kg/m^3^-12 kg/m^3^.

**Fig. 6:**
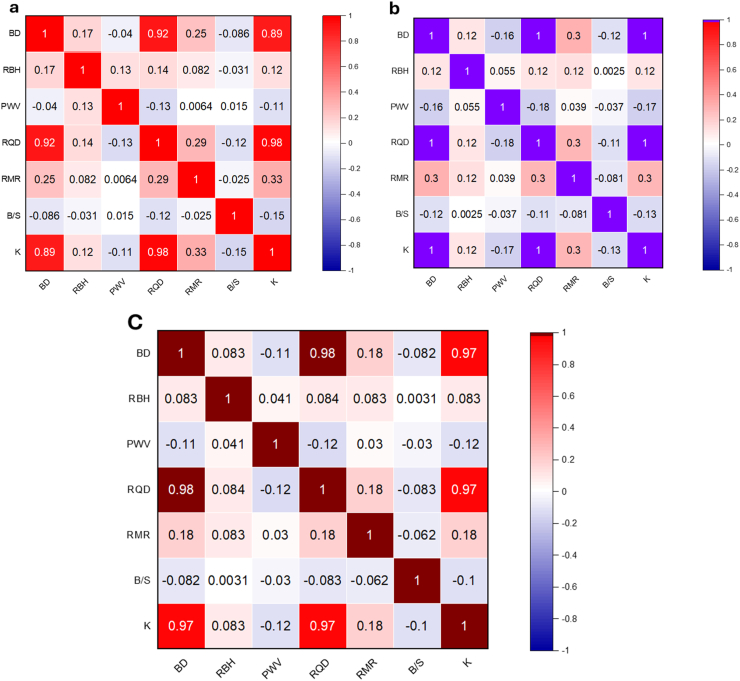
The illustration of correlation plot heat maps based on (a) Pearson, (b) Spearman and (c) Kendall correlation.

The Pearson correlation coefficient, which has a range of −1 to 1, is frequently used to evaluate the relationship between two variables. A correlation value of 0 indicates no relationship between the variables, while a positive correlation coefficient indicates a positive relationship among the variables, and a negative correlation coefficient indicates a negative relationship between the variables. Furthermore, the degree of correlation may be derived from the magnitude of the correlation coefficient, where a greater absolute number signifies a stronger correlation between the variables. Fig. 6a Pearson correlation plot demonstrates a strong positive relationship between RQD and BD, whereas B/S, RBH, and BD demonstrate a negative correlation. It should be emphasized that Pearson's coefficient can only provide an overview of the linear correlation between two variables.

Pearson's coefficient may not adequately represent non-linear interactions. Hence, it is essential to analyze the outcomes of the correlation analysis within the framework of the variables under investigation, together with any possible non-linear relationships that may be present. To establish a non-linear correlation between the variables, Spearman correlation (see Fig. 6b, and Kendell correlation (see Fig. 6c**)** were used. More precisely, it quantifies the extent to which information about one variable may be acquired by monitoring the other variable. Both non-linear correlation techniques can capture non-linear correlations, handling both categorical and continuous variables. Fig. 6b clearly demonstrates that the variables RQD and RMR have the greatest values correlation. This discovery is notable since it is not distinguishable from the findings obtained using the Pearson correlation coefficient approach.

**Fig. 7:**
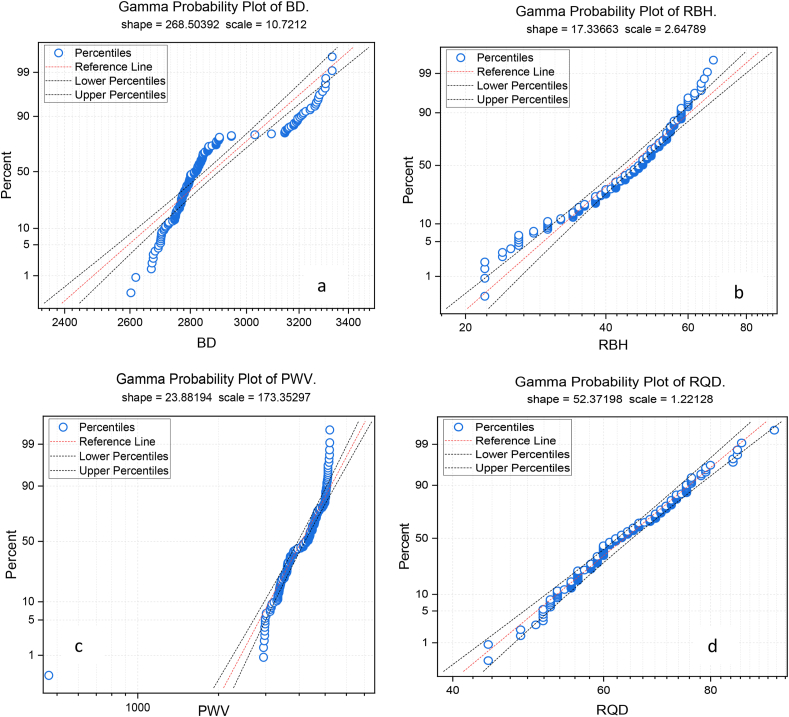

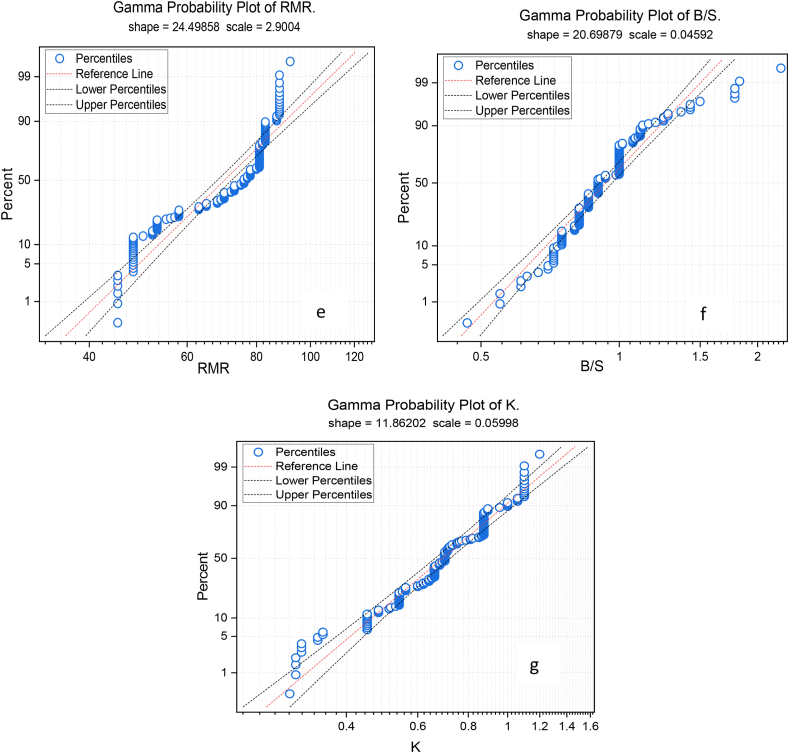
The illustration of probability P–P plot of all variables.

During the data analysis stage, researchers use the probability (PP) plot to visualize dataset dispersion. The PP plot compares real data quantiles to predictions of a theoretical distribution, revealing the data's distribution. This graph helps determine whether data follows a probability distribution like the normal distribution. Variations from a linear trajectory in the PP plot may indicate variations from the anticipated distribution, highlighting dataset issues. For this current study, the PP plot investigates each variable (see Fig. 7 a-g**);** based on the PP plot result, variables such as RQD and RBH show almost good distribution in terms of the typical pattern of the data; however, some of the parameters show different from the normal distribution. Essentially, the specified μ and σ in the typical probability plot provide a reference for assessing the normality of the dataset under consideration.

## 4 Model development methodology

### 4.1 Artificial neural network algorithm for powder factor prediction

An ANN is a computer model that draws inspiration from the neuronal organization of the human brain [63]. These strategies involve the use of artificial intelligence to create models. ANNs consist of interconnected nodes, known as neurons, that are arranged in layers. ANNs can learn and make judgments based on data. The input nodes receive the input data, which is then processed by the hidden layers of the ANN through weighted connections. Finally, the output nodes generate the results. Fissha [64] elucidated that the training of ANN entails the fine-tuning of weights in accordance with the network's performance in achieving desired outcomes, with the objective of maximizing accuracy. Several authors have embraced this strategy over the years. Gebretsadik [65] employed an artificial neural network (ANN) model and compared it with other machine learning approaches to predict rock fragmentation. He concluded that an ANN model with an architecture of 5-64-32-16-1 demonstrates superior performance. Ahmadi [66] employed ANN to model the performance of oil well production. The created model incorporates methodologies that may be integrated into numerical reservoir simulation software to enhance accuracy and improve parametric sensitivity analysis. This study aimed to construct a powder factor model by considering six input parameters: bulk density, rock mass rating, rebound hardness value, burden-to-spacing ratio, rock grade designation, and P-wave velocity. The ANN model was constructed using Bayesian regularization (MD1) and Levenberg-Marquardt (MD2) training techniques with a 6-5-1 architecture. Table 1 displays an overview of the database's descriptive statistics. It includes the input factors of the ANN dataset that have been found in the literature to be influential on the powder factor.

**Table 1:** Descriptive statistics of the dataset.

Descriptive statistics	BD	RBH	PWV	RQD	RMR	B/S	K
Mean	2878.68	45.91	4140.01	63.96	71.06	0.95	0.71
Median	2817.99	47.50	4241.00	62.50	76.00	0.90	0.70
Mode	2863.63	50.00	4484.00	60.00	81.00	1.00	0.87
SD	180.42	10.37	736.16	8.96	13.63	0.23	0.20
Kurtosis	0.30	−0.40	1.84	0.21	−1.03	8.14	−0.24
Skewness	1.25	−0.41	−0.71	0.50	−0.62	2.19	0.11
Range	730.16	46.00	4742.00	51.00	47.00	1.78	0.91
Minimum	2603.17	22.00	466.00	44.00	45.00	0.47	0.29
Maximum	3333.33	68.00	5208.00	95.00	92.00	2.25	1.20
Sum	518163.23	8263.0	745201.0	11513.0	12790.0	171.08	128.06
Count	180.00	180.00	180.00	180.00	180.00	180.00	180.00

One hundred forty-four datasets were used to train the neural network in the MATLAB© environment using the nnstart toolbox. The input and output variable elements were first normalized between −1 and 1 using Eq. (4) to achieve dimensional consistency in the variable elements and also to eliminate over-fitting.

(4) $$Xi=\frac{2\left(Yi-Ymin\right)}{\left(Ymax-YmIN\right)}-1$$

where Xi is the normalized data, Yi is the actual data to be normalized, *Y*_max_ and *Y*_min_ are the maximum and minimum values of the actual data, respectively. Thirty-six out-of-state datasets were used to test the developed ANN model. The MD1 and MD2 were compared using mean square error and coefficient of correlation (R^2^) (See Table 2). The optimum model hyperparameters were de-normalized using Eq. (5) and converted to mathematical equations.

(5) $$Yi=\left(\frac{Ymax-Ymin}{2}\right)Xp+\left(\frac{Ymax+Ymin}{2}\right)$$

where X_P_ is the predicted elements, *Y*_*i*_ is the actual predicted data de-normalized, *Y*_max_ and *Y*_min_ are the maximum and minimum values of the actual data, respectively.

**Table 2:** Comparison between two developed ANN models.

	MD1		MD2	
	Train	Test	Train	Test
**R**^**2**^	0.958	0.944	0.985	0.962
**MSE**	0.00228	0.00189	0.000496	0.00107

**Fig. 8:**
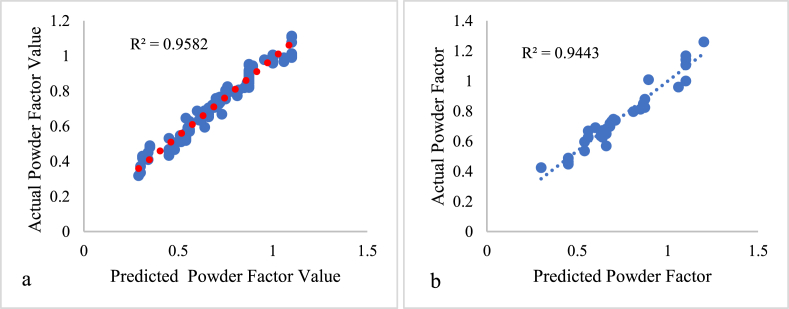
Relationship between the predicted and actual powder Factor value for MD1; A: Training data, B: out-of-state testing data.

**Fig. 9:**
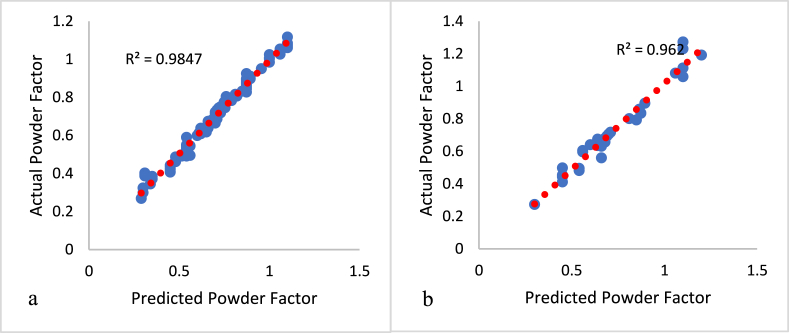
Relationship between the predicted and actual powder Factor value for MD2; A: Training data, B: out-of-state testing data.

The model results are presented in Fig. 8, Fig. 9 demonstrate that for training and out-of-state testing data, MD 1 had a coefficient of correlation (R^2^) of 0.958 and 0.944, while for MD 2, the values were 0.985 and 0.962, respectively, when comparing the two models' predicted results with the actual powder factor values. Fig. 8, Fig. 9a present the relationship between the actual and predicted powder factor from the training phase for both MD1 and MD2. While, Fig. 8, Fig. 9b present the relationship between the actual, model 1 (MD1) and model 2 (MD2) predicted powder factor result for the out of state testing dataset respectively.

The MD 2 with Bayesian regularization was found to be more robust than Levenberg-Marquardt. According to Burden and Winkler [67], it was discovered that the MSE value in Table 2 demonstrated the elimination of the requirement for extensive cross-validation. The MD1 ANN model was translated into a mathematical equation to forecast the powder factor. The expression is given in Eq. (6).

(6) $$K=0.405\;\tanh\;\left(\sum_{i=1}^{5}N_{i}\;-\;0.6964\right)+0.695$$

$$N_{1}=1.2369\;\tanh\left(-1.0028\text{BD}+0.20145\text{RH}-0.1035\text{PV}+0.12649\;\text{RQD}+1.1654\text{RMR}-0.4652B/S+0.6937\right)$$

$$N_{2}=1.5124\;\tanh\left(1.7865\text{BD}+0.0971\text{RH}-0.0335\text{PV}+0.0269\;\text{RQD}+0.1283\text{RMR}+1.67107B/S+0.0790\right)$$

$$N_{3}=-0.8678\;\tanh\left(0.3883\text{BD}-0.0402\text{RH}-0.01773\text{PV}-0.9198\;\text{RQD}+0.4010\text{RMR}-0.3057B/S-0.7031\right)$$

$$N_{4}=-0.8847\;\tanh\left(0.5788\text{BD}+0.2640\text{RH}-0.02897\text{PV}-0.4651\;\text{RQD}+1.3922\text{RMR}-0.1870B/S-0.3874\right)$$

$$N_{5}=-1.0556\;\tanh\left(0.0944\text{BD}+0.1906\text{RH}-0.1154\text{PV}+0.1550\;\text{RQD}+0.0214\text{RMR}+2.2257B/S+1.3577\right)$$

where K is the powder Factor in kg/m^3^, BD is bulk density in kg/m^3^, RH is the rebound hardness value, PV is the P-wave velocity in m/s, RQD is the rock quality designation, and RMR is the rock mass rating.

This paper explores the use of ANN as a novel method for predicting powder factors by extracting equations. This study utilizes an ANN approach to identify complex patterns and non-linear relationships in data related to rock strength, classification system, induced wave response effect, and discontinuity property base system. Unlike traditional methods, the ANN approach is able to capture intricate dependencies that conventional models may miss. The ANN model in this study creates its internal representation of the equation that governs the powder factor, enabling it to adjust and acquire knowledge from various datasets. For precise powder factor forecasting, the ANN model proposed in this study is a potent tool due to its versatility and capacity to handle complex rock characteristic interactions, especially in mining situations where the underlying dynamics are complex and challenging to model using conventional equations.

### 4.2 Gaussian product regression (GPR) algorithm for powder factor prediction

The GPR algorithm is a potent tool in the domain of machine learning, particularly in regression analysis [68]. The GPR algorithm, as explained by Gbadamosi [69] and Harkat [70], relies on the capability to represent the connection between input parameters and the target variable through a Gaussian distribution. They stated that the target variable in this technique is thought to be a linear combination of weighted Gaussian basis functions, each with a unique weight coefficient. During the modeling phase, the input features define the basis of GPR functions, which represent the underlying patterns in the data.

The GPR algorithm seeks to determine the weight coefficients and hyperparameters of the Gaussian basis functions by maximizing the likelihood of the observed data. Researchers employ an iterative optimization approach, such as maximum likelihood estimation or Bayesian inference, to accomplish this goal. The ultimate model may thereafter forecast the target variable for novel input characteristics by utilizing a fusion of the weighted basis functions. Using the GPR algorithm in constructing a powder factor regression model has numerous benefits. According to Wudil [71], the strategies can be modeled in a flexible and non-linear manner, enabling them to accurately represent intricate relationships between the input data and the target variable.

**Fig. 10:**
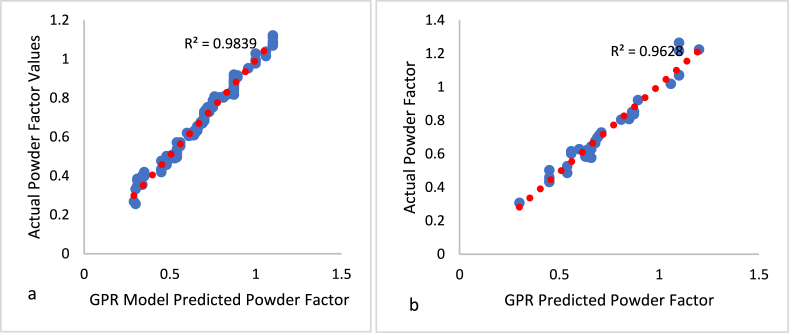
Relationship between the predicted and actual powder Factor value for MD3; A: Training data, B: out-of-state testing data.

In addition, GPR, utilizing its probabilistic framework, offers uncertainty estimates for its predictions and can effectively handle outliers, making it a powerful tool in decision-making processes [72]. The GPR model (MD3) proposed in this study consists of one dependent variable, namely a powder component, and six independent variables, as shown in Table 2. The GPR model was trained using 80 % of the dataset and assessed using a 20 % portion of the normalized database in a MATLAB© environment. The GPR hyperparameters, including kernel scale, kernel function, basic function, and sigma, were automatically determined according to the methodology outlined in Fissha [73]. The model's prediction was compared to the output of the training dataset and is displayed in Fig. 10a, showing an R^2^ value of 0.984. The model was tested using data from outside the state, and the correlation graph is displayed in Fig. 10b, indicating a coefficient of determination (R^2^) of 0.962.

### 4.3 Decision and ensemble tree algorithm for powder factor prediction

**Fig. 11:**
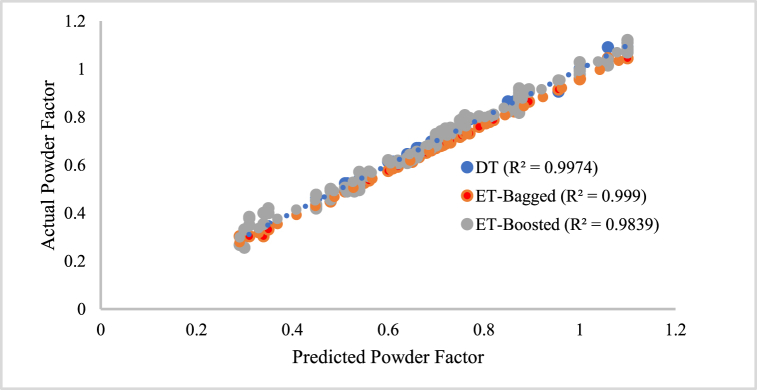
Relationship between the predicted and actual powder Factor value for MD4, MD5, and MD6 on Training data.

**Fig. 12:**
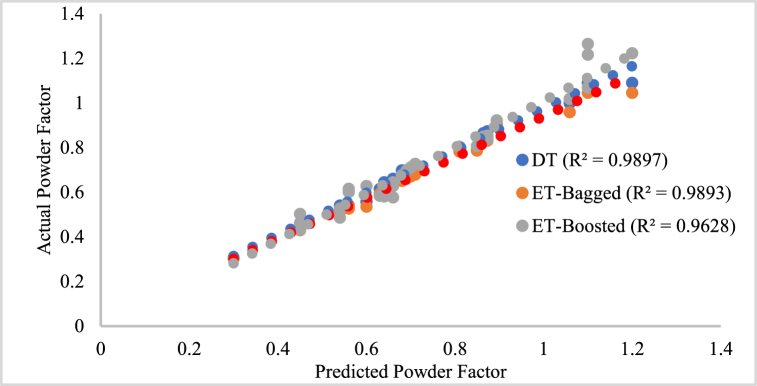
Relationship between the predicted and actual powder Factor value for MD4, MD5, and MD6 on Test data.

Machine learning has widely used DT algorithms to solve classification and regression problems. According to Costa and Pedreira [74], decision tree algorithms provide a systematic and intuitive way of making predictions by splitting the data into branches based on the values of its features. The algorithm makes the final prediction by following a path through the tree, with each branch representing a different outcome or class label. Bollwein [75] explained that the decision tree algorithm works by recursively partitioning the data based on the feature values that maximize the information gain or minimize the impurity at each splitting node. During the modeling stage, the process continues until a stopping criterion is met, such as reaching a maximum depth or having a minimum number of samples in a node. The resulting tree can then be used to make predictions by traversing the tree from the root to a leaf node, following the decision rules at each node. He et al. [76] used a decision tree to predict air overpressure generated from blasting operations as a way forward to minimizing environmental challenges attributed to mining. This study ran the DT model (MD4) configuration for powder factor prediction with 30 iterations, within 310s of the maximum training time, and a Bayesian optimizer. Another popular tree algorithm used for powder factor optimization is the RF, which constructs an ensemble of decision trees by randomly selecting subsets of the training data and features for each tree. The final prediction is obtained by averaging the predictions of all the individual trees. This randomness in the training process helps to reduce overfitting and improve the generalization performance of the model. A wide range of machine-learning tasks have proven the effectiveness of both bagged and boosted random forests, powerful ensemble tree algorithms [77,78]. These modeling techniques offer a balance between model complexity and generalization performance, making them suitable choices for various real-world applications. The proposed DT model (MD4), bagged RF model (MD5), and boosted RF model (MD6) all have one dependent variable, which is a powder component, and six independent variables, which can be seen in Table 2. The three models (MD 4, MD 5, and MD 6) were trained using 80 % of the dataset and assessed using a 20 % portion of the normalized database in a MATLAB© environment. The model's prediction was compared to the output of the training dataset, and the training and testing phase results are displayed in Fig. 11 and. 12. The model testing result using data from outside the state shows a coefficient of determination (R^2^) of 0.9897, 0.9893, and 0.9628 for MD4, MD5, and MD6, respectively (see Fig. 12).

### 4.4 Support vector regression algorithm for powder factor prediction

The SVR technique is a robust and effective method in the field of machine learning, specifically designed to address regression problems [79]. The approach expands upon the SVM algorithm, which is usually utilized for classification work. Regression aims to forecast a continuous numerical outcome by utilizing a collection of input characteristics. According to Basak [80], SVR addresses this issue by identifying the optimal hyperplane that effectively separates the data points in a space with many dimensions. The approach of SVR modeling offers the capability to effectively manage many forms of loss functions, including epsilon-insensitive loss and squared loss. Suradhaniwar [81]stated that SVR employs Epsilon-insensitive loss, which permits a specific tolerance around the predicted value.

In contrast, squared loss imposes more penalties on higher mistakes. SVR, in terms of computing efficiency, depends on a specific subset of training data known as support vectors. These support vectors are the data points that are positioned closest to the hyperplane. SVR is able to efficiently process big datasets by selectively analyzing a portion of the data during both training and prediction phases.

**Fig. 13:**
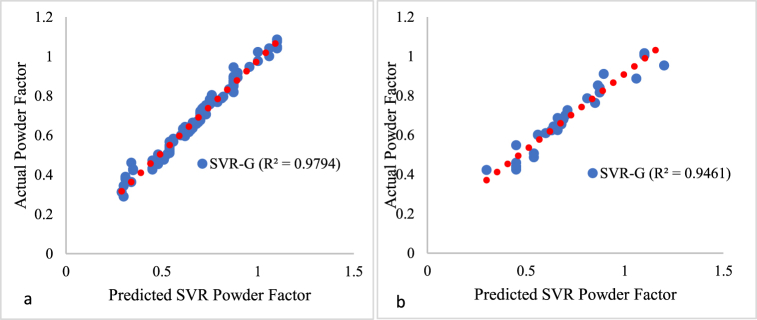
Relationship between the predicted and actual powder Factor value for MD7 on Training and Test data.

The SVR model (MD7) proposed in this study consists of one dependent variable, namely a powder component, and six independent variables, as shown in Table 2. The MD7 was trained using 80 % of the dataset and assessed using a 20 % portion of the normalized database in a MATLAB© environment. The model's prediction was compared to the output of the training dataset, and the training and testing phase results are displayed in Fig. 13 (a and b). The model training (Fig. 13 a) and testing (Fig. 13b) result shows the coefficient of determination (R^2^) of 0.9794 and 0.9461, respectively.

## 5 Model performance evaluation

The prediction accuracy of the proposed artificial intelligence model for powder factor prediction in this study was assessed using five statistical metrics: root mean square error, mean absolute error, coefficient of determination, mean absolute percentage error, variance accounted for, weighted mean absolute percentage error, and performance index. These indices are specified in Eqs. (7), (8), (9), (10), (11) [82].

(7) $$R^{2}=1-\left(\frac{\sum_{i=1}^{n}{\left(O_{i}-P_{i}\right)}^{2}}{\sum_{i=1}^{n}{\left(P_{i}-{\bar{P}}_{i}\right)}^{2}}\right)$$

(8) $$RMSE=\sqrt{\frac{1}{n}\sum_{i=1}^{n}{\left(O_{i}-P_{i}\right)}^{2}}$$

(9) $$VAF=100\cdot \left(1-\frac{var\left(O_{i}-P_{i}\right)}{var\left(O_{i}\right)}\right)$$

(10) $$MAE=\frac{1}{n}\sum_{i=1}^{n}\left|\left(P-O\right)\right|$$

(11) PI=R2+(VAF100)−RMSE

Where *O*_*i*_ signifies measured value, *P*_*i*_ indicates predicted value, ${\bar{P}}_{i}$ and $\bar{O}$ are average of the estimated and actual values, respectively; *n* signifies the number of data sets.

Additionally, the study of the model's performance was conducted using the Final Rating (FR) approach as present in Eq. (12). The FR approach was employed to allocate ratings to all the findings of model evaluation induces. The model that exhibited the lowest RMSE, MAE, and WMPE values, as well as the highest R^2^, Performance Index (PI), and Variance Accounted For (VAF) values, among other metrics, was assigned a higher rank. The ranking is contingent upon the number of developed models generated for each technique.

(12) $$FR=\sum_{i=1}^{2}\left(r_{i}^{R^{2}}+r_{i}^{RMSE}+r_{i}^{VAF}+r_{i}^{MAE}+r_{i}^{BF}\right)$$

where *r*_*i*_ indicates the rate of evaluation criteria, *i* stands 1 for train rates of evaluation indicators or 2 for test rates of evaluation indicators.

### 5.1 Powder factor model performance discussion

The RSME measures how close the expected and observed powder factors are to each other, while the MAE measures how big the difference is between the actual and predicted values from the models. A drop in the root mean square error (RSME) and mean absolute error (MAE) signifies a decrease in the difference between the actual powder factor and the anticipated values. Furthermore, R2 and VAF serve as indicators of the suggested model's accuracy level compared to the actual measured powder factor values. The optimum model for powder factor forecasting in small-scale mine blasting operations was selected in this study based on their low RSME and MAE values, as well as their high R2 and VAF values. Table 3, Table 4 present the errors investigated in this study. The results show that the decision tree model (MD4) does better than the MD1, MD2, MD3, MD5, MD6, and MD7 models when it comes to RSME and MAE, having the lowest values for both. Based on the correlation coefficient study, the decision tree model MD4 outperforms the artificial neural network methods (MD1 and MD2) by 4.77 % and 3.02 %, respectively. The MD4 algorithm outperforms the GPR method (MD3) in correctly forecasting powder factor values using rock properties, resulting in a notable improvement of 2.81 %. The model exhibited improved performance in comparison to the bagging and boosting tree models (MD5 and MD6), with respective increases of 0.92 % and 3.02 %. The comparison between the DT model and the support vector regression model reveals that the DT model outperforms the support vector regression model by 4.55 %, as shown in Table 3, Table 4 We used the mean deviation squared (MD2) method to find the best artificial neural network (ANN) equation for figuring out the best powder factor value for small-scale blasting operations. The mathematical equation of the Artificial Neural Network (ANN) demonstrates a significant level of accuracy in predicting the powder factor value.

**Table 3:** Model evaluation analysis results.

				Training Data			
	MD1	MD2	MD3	MD4	MD5	MD6	MD7
R^2^	0.958	0.985	0.984	0.997	0.999	0.984	0.979
RSME	0.046164	0.0237	0.0243	0.009853	0.0318	0.0243	0.0288
VAF	95.2	98.5	98.4	99.7	99.71	98.38	97.7
MAE	0.036	0.0178	0.0195	0.0057	0.0303	0.0195	0.0228
PI	1.863836	1.9463	1.9437	1.984147	1.9643	1.9435	1.9272

				Testing Data			
	MD1	MD2	MD3	MD4	MD5	MD6	MD7
R^2^	0.944	0.96	0.962	0.989	0.989	0.96	0.946
RSME	0.0031	0.0023	0.00212	0.00067	0.0207	0.002123	0.00481
VAF	94.39	95.23	95.66	98.77	98.34	95.66	91.39
MAE	0.044	0.0316	0.0328	0.0147	0.0367	0.0328	0.0463
PI	1.8848	0.957721	1.91648	1.97603	1.9517	1.914477	1.85509

**Table 4:** Model evaluation ranking analysis result.

			Training Data				
	R^2^	RSME	VAF	MAE	PI	Sum	Ranking
MD1	1	1	1	1	1	5	7
MD2	5	6	5	6	5	27	2
MD3	3	4	4	4	3	18	5
MD4	6	7	6	7	7	33	1
MD5	7	2	7	2	6	24	3
MD6	4	5	3	5	4	21	4
MD7	2	3	2	3	2	12	6

			Testing Data				
	R^2^	RSME	VAF	MAE	PI	Sum	Ranking
MD1	1	3	2	2	3	11	6
MD2	3	4	3	6	1	17	5
MD3	5	6	4	4	5	24	2
MD4	6	7	7	7	7	34	1
MD5	6	1	6	3	6	22	3
MD6	3	5	4	4	4	20	4
MD7	2	2	1	1	2	8	7

### 5.2 Sensistivity analysis

**Fig. 14:**
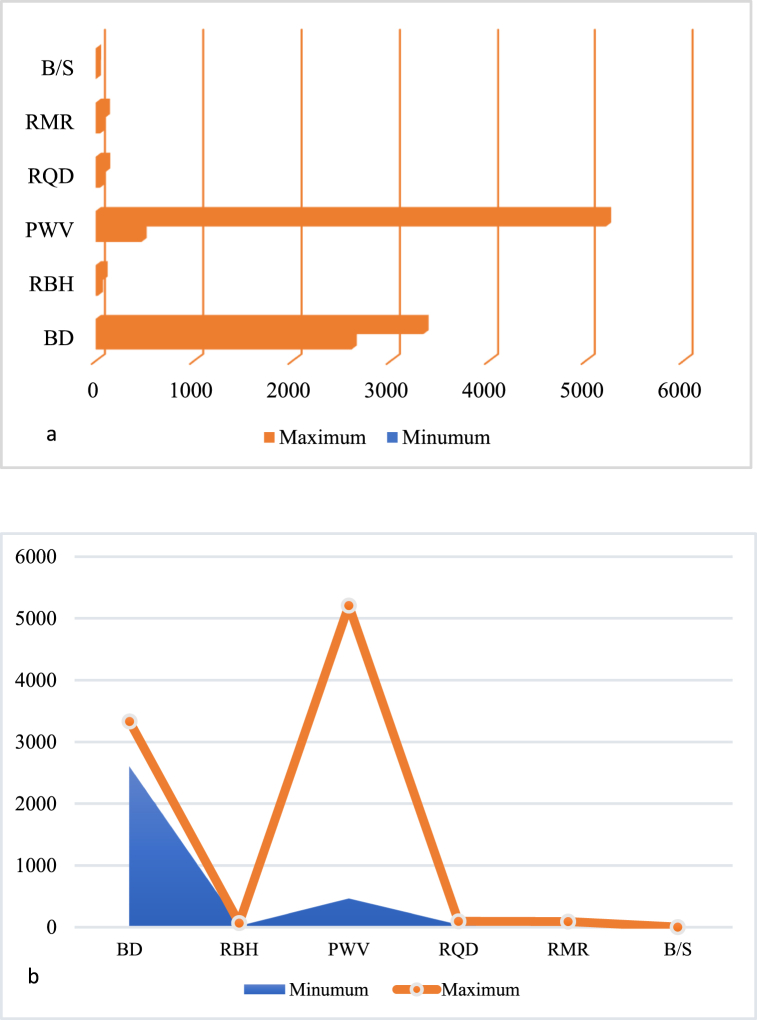
Sensitivity analysis of the study using Tornado analysis.

Tornado input parameters impact the outcomes of a model or simulation [83]. Tornado sensitivity analysis shows how the output of the developed model changes based on the input variables. This gives information on the most important factors, which makes the results more reliable and stable. We employed tornado sensitivity analysis in this study to assess the influence of various input parameters on our model output (powder factor), and found that the rock P-wave significantly affects the outcomes at a large percentile value, exerting a substantial impact. Conversely, we observed that the burden-to-spacing (B/S) ratio, which defines the explosive energy distribution, has a comparatively lesser influence on the model. This analysis enabled us to prioritize our focus on refining the parameters with the greatest impact on the P-wave in order to improve the accuracy and reliability of our research findings. This analysis by Zhang [84] supports the findings that the speed of P-waves through rock influences the choice of explosive required, as it determines the density and strength of the rock, which are crucial factors in selecting the appropriate explosive for efficient fragmentation. Fig. 14 (a and b) illustrates the Tornado sensitivity analysis plot for all the input variables.

### 5.3 Comparison of excavator productivity before and after optimum model application

**Fig. 15:**
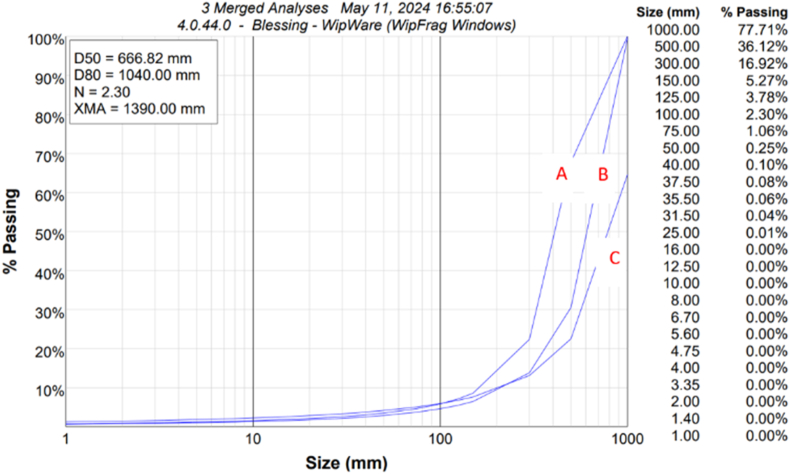
Blast Fragmentation result for three merged blast; A&B: optimized blast; C: Blast before optimization.

In the case study with two blast designs, we applied the optimum model to two new blasts. To optimized the burden-to-spacing ratio parameter using MD 4 to determine the optimal powder factor for the mine, taking into account the uncontrollable factors. The blast was assessed using the D50, uniformity index, and D80 fragmentation sizes. The blast results was assessed using WipFrag 4 software (available here: https://wipware.com/get-wipfrag/) and used a 0.5 m by 0.5 m scaling object to analyze the blast muck pile. We optimized the blast fragmentation from 864.33 mm to 666.83 mm (22.8 %) for mean size and from 1135.8 mm to 1040 mm (8.43 %) for 80 % passing size. Fig. 15 and Table 5 present the MD 7 model simulation results.

**Table 5:** Comparison of blast fragmentation before and after MD 4 model application.

Blast design			Blast Size Distribution statistics					
	Before	After						
B (m)	2	1.8	X_50_		X_80_		n	
S (m)	2	2.25	Before	After	Before	After	Before	After
T (m)	1.2	1.2	864.22 mm	666.82 mm	1135.8 mm	1040 mm	2.2	2.3
H (m)	8.5	8.5						
CL (m)	7.2	7.2						
PF (kg/m^3^)	0.9	0.85						

## 6 Conclusion

Utilizing machine learning approaches to forecast powder factors based on essential rock parameters shows potential for improving explosive use efficiency in mining operations. This study utilized a database of small-scale mine blast operations in south-south Nigeria.

In conclusion, the Pearson correlation coefficient analysis on the database contains RMR, RQD, rock density, P-Wave velocity, and B/S. Rebound hardness offers valuable information about linear associations between the proposed model variables and also detects non-linear interactions. By employing Spearman and Kendall correlations, this study identified meaningful connections, particularly between RQD and RMR.

A strong positive association (r = 0.88) was observed between powder factor and bulk density. The indicated increase in bulk density suggests that the rock is more densely packed, resulting in a greater amount of energy needed to fracture it. This effect of this is seen in an increased powder factor. The correlation between the findings highlights the need to understand rock density characteristics to optimize blasting designs and achieve the desired fragmentation results in mining and quarrying operations.

The findings of the ANN model demonstrate the efficacy of machine learning techniques in forecasting powder factor values for mining blasting operations. When comparing the MD1 and MD2 models, it is evident that the MD2 model outperforms the MD1 model in terms of performance metrics. Specifically, the MD2 model shows higher values for the coefficient of correlation and error indices, indicating greater accuracy in both the training and out-of-state testing data sets. Furthermore, the implementation of Bayesian regularization in MD2 improves its resilience in comparison to the Levenberg-Marquardt algorithm. Converting the MD1 ANN model into a mathematical equation offers a useful forecasting tool for estimating powder factors.

Finally, the overall error analysis conducted on the seven models for forecasting powder factor in small-scale mine blasting operations revealed that the decision tree model (MD4) emerged as the most effective, exhibiting the lowest RSME and MAE values. Moreover, MD4 demonstrated superior performance compared to other models, including artificial neural networks (MD1 and MD2), Gaussian process regression (MD3), bagging and boosting tree models (MD5 and MD6), and support vector regression.

The results underscore the significance of accurate modeling in optimizing powder factor selection, with the MD4 algorithm providing a reliable method for predicting powder factor values based on rock parameters. The developed model was applied to three blasts, and the fragmentation was optimized from 864.33 mm to 666.83 mm (22.8 %) for the mean size and 1135.8 mm–1040 mm (8.43 %) for the passing size. Additionally, the created artificial neural network equation provides a precise way to figure out the best powder factor for small-scale blasting operations, which leads to safer and more efficient mining practices.

## Ethical statement

The authors state that the research was conducted according to ethical standards.

## Funding body

This research received no external funding.

## Data availability

The data used in this study is available at reasonable request from the corresponding author.

## CRediT authorship contribution statement

**Blessing Olamide Taiwo:** Writing – original draft, Software, Resources, Methodology, Investigation, Formal analysis, Data curation. **Angesom Gebretsadik:** Methodology, Investigation, Formal analysis, Data curation. **Hawraa H. Abbas:** Visualization, Validation. **Mohammad Khishe:** Writing – review & editing, Validation, Supervision, Project administration, Methodology, Conceptualization. **Yewuhalashet Fissha:** Software, Resources. **Esma Kahraman:** Validation, Investigation. **Ahsan Rabbani:** Visualization, Validation. **Adams Abiodun Akinlabi:** Writing – review & editing, Validation.

## Declaration of competing interest

The authors declare that they have no known competing financial interests or personal relationships that could have appeared to influence the work reported in this paper.
